# History and Social Implications of the Pulse Oximeter

**DOI:** 10.7759/cureus.68250

**Published:** 2024-08-30

**Authors:** Dean K Zacharis, Daniel Z Zhao, Latha Ganti

**Affiliations:** 1 Biomedical Sciences, University of Central Florida, Orlando, USA; 2 Biology, Brown University, Providence, USA; 3 Emergency Medicine and Neurology, University of Central Florida, Orlando, USA; 4 Research, Orlando College of Osteopathic Medicine, Winter Garden, USA; 5 Medical Science, The Warren Alpert Medical School of Brown University, Providence, USA

**Keywords:** healthcare disparities, oxygen saturation, vital signs, medical education, pulse oximetry

## Abstract

The pulse oximeter is a portable, bedside tool that allows for the measurement of oxygen saturation in a patient's red blood cells. The technology is based on oxygenated and deoxygenated hemoglobin absorbing light at different wavelengths. The device calculates the ratio of oxygenated to deoxygenated hemoglobin in the blood, and an algorithm produces a percentage oxygen saturation value. Due to its portability and ease of use, it is a ubiquitous medical tool that is commonly used in medical practice. This paper reviews the history and evolution of this tool, and the scientific laws behind oximetry. It also introduces the importance of the pulse oximeter and its basic functions. In addition, the limitations of pulse oximetry are discussed, especially as they pertain to pigmented skin.

## Introduction and background

The pulse oximeter is a revolutionary medical device used globally by healthcare practitioners. This device measures the amount of oxygen in a patient’s blood, known as oxygen saturation [1]. On the pulse oximeter, arterial oxygen saturation is depicted by SpO₂, shown as a percentage [2,3]. This percentage represents the percentage of hemoglobin, the protein found in red blood cells that carries oxygen from the lungs to the rest of the body, that is oxygenated. Generally, the SpO₂ level of a healthy individual ranges between 95% and 100%. This range is the benchmark most healthcare centers and practitioners use when reading a pulse oximeter. The SpO₂ is an essential vital sign that aids in monitoring patients within the hospital [4]. Specifically, the pulse oximeter can detect hypoxia relatively early, allowing for early action and treatment [3]. What makes the pulse oximeter revolutionary is its convenient, portable, and compact design [5]. Although there are different pulse oximeters, from the fingertip pulse oximeter to the ear pulse oximeter, they are generally straightforward to use, making them quite reputable in the medical world. Moreover, they are easily accessible over the counter to individuals, encouraging the overall use of pulse oximeters at home. This accessibility could help unveil the possibility of an individual having hypoxia without needing to seek aid at a medical facility [6]. The modern-day pulse oximeter is well-refined over centuries of technological innovation and development. The pulse oximeter’s reputation is well supported by studies finding an increase in its usage and positive perception of the pulse oximeter as a helpful instrument in the medical world [4].

However, the modern-day pulse oximeter is much different than the one 100 years ago. Initially, measuring oxygen saturation in the blood traces back to the 1800s when hemoglobin was first isolated [7]. This discovery led to further work by researchers to use light absorption laws from the 1700s to develop a method to measure oxygen saturation in the blood. Subsequently, the 1900s was a significant period of research for the pulse oximeter, specifically fueled by major events such as World War Two, to reduce hypoxia in pilots [8]. Ultimately, it was not until 1972 that Takuo Aoyagi introduced the concept of “pulse” in oximetry. His integration of arterial pulse in oximetry directly led to the innovation that allowed for accurate blood oxygen saturation measures [9]. Takuo Aoyagi made a groundbreaking discovery that gave rise to a new definition for the modern-day pulse oximeter, however, it is not faultless. Although tremendous time and effort have been put into innovating this device, the pulse oximeter still has limitations. A primary concern for many is the inaccuracies of the pulse oximeter depending on one’s skin pigmentation [10]. The inaccuracy is relatively minimal, but in the medical world, this difference may determine whether or not a patient is diagnosed with hypoxia. However, this is not to discredit the evolution and development of the modern-day pulse oximeters, as they have been universally integrated within the medical field and, despite their flaws, are still effective.

## Review

**Figure 1:**
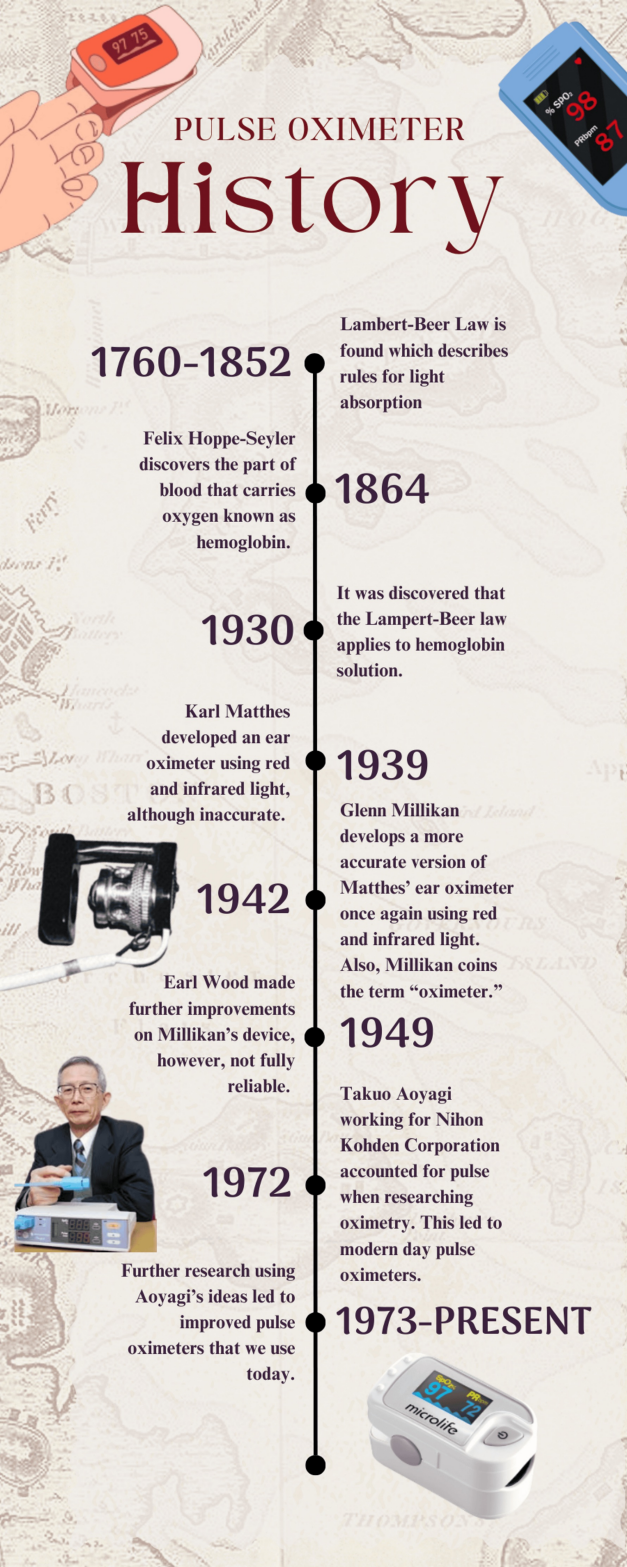
Infographic depicting the history of the pulse oximeter. Designed by Dean Zacharis on Canva.com

Primarily, simple oximetry itself measures how much hemoglobin is saturated with oxygen in the blood using the relationship between light transmission and absorbance [7]. This type of oximetry is the predecessor to modern-day pulse oximetry and is derived from the Lambert-Beer law. Scientific research dating back to Johann H. Lambert in 1760 applies to oximetry because pulse oximetry uses light absorption to measure the oxygen saturation within the blood. This phenomenon is why oximetry uses Lambert’s law, describing how an object’s thickness is proportional to how much light it absorbs [7]. Even though this law was not specific to oximetry or even medicine, it is an integral law that allows for oximeters to calculate absorption and thus calculate how much oxygen saturation there is in the blood. Furthermore, August Beer built upon Lambert’s discoveries in 1852 by describing the proportional relationship between absorption and the concentrations of the attenuating species within the material sample [7]. These two discoveries together form the Lambert-Beer law, which is the basis of all oximetry (Figure 1) [11-13].

Next, the invention of the spectroscope in 1860 by Gustav Kirchhoff and Robert Bunsen was the next step toward creating an accurate way to measure oxygen saturation [7]. The spectroscope allows for the analysis of the spectra of any chemical compounds. Combined with the isolation of hemoglobin in 1864 by Felix Hoppe-Seyler, the spectroscope is an essential tool in the history of the pulse oximeter [14]. Felix Hoppe-Seyler used the spectroscope to study hemoglobin and found that hemoglobin absorbed two different wavelengths of light: 560nm and 540nm [7]. After discovering these wavelengths, George G. Stokes experimented with hemoglobin and Hoppe-Seyler’s work to find that the two wavelengths of hemoglobin vanished as the blood became deoxygenated [7]. This finding proved that hemoglobin could exist in an oxygenated and a deoxygenated state. Additionally, in the 1930s, the Lambert-Beer law was found to be applicable to oxyhemoglobin solutions. Consequently, many set out to use the Lambert-Beer law and newfound knowledge about hemoglobin to find an accurate way to measure blood oxygen saturation.

The first attempt at an oximeter in the early 1900s consisted of removing blood from the patient's test site, recording the result, then allowing the blood to flow back to the area and comparing the two readings [7]. Although this is similar to modern-day pulse oximeters, it could have been more efficient and could not be precise every time. One of the first attempts at an oximeter was Karl Matthes’ oximeter, built in 1939 and attached to the ear [8]. Matthes’ oximeter used red light absorbed by oxyhemoglobin and infrared light, which he labeled the “green” light. However, this oximeter was inaccurate in practice because it was hard to calibrate, did not accommodate changes in pulsatile flow, and the light would get absorbed by other tissues instead of the arterial flow [7]. Even further progress was made when J.R. Squire used pneumatic pressure to mitigate the effects of background light in oximetry [7]. In 1942, Glenn Millikan took advantage of Squire’s research and combined it with Matthes' earlier oximeter [14]. This new oximeter was more accurate than Matthes’ oximeter [15]. Later, Earl Wood changed the previous green light filter into an infrared light, which improved efficacy. Still, his device was not optimal at detecting the changes in light levels, leading to inaccurate readings [7,8].

Despite all the recent progress in oximetry, Kramer and J. Elam discovered that Beer’s law was misapplied to oximetry [7]. Afterward, a new oximeter was developed and sold in the 1970s using eight wavelengths instead of two. This oximeter was convenient because it did not require a bloodless area to measure oxygen saturation, unlike its predecessors [7]. However, this device was expensive and too big to be applied effectively throughout the medical world. In 1972, Takuo Aoyagi revolutionized oximetry by accommodating pulsation, which interfered with accurate readings [9]. Aoyagi worked to eliminate the interference of this pulsatile “noise”. He successfully used ratios to cancel out pulsatile noise thus making the first pulse oximeter [8]. This innovation set the stage for further development of accurate pulse oximeters, which led to the modern pulse oximeter we have today.

Between 1973 and the present, a period of rapid progress for the pulse oximeter began. Many companies used Aoyagi’s research to develop marketable pulse oximeters [7]. Indeed, pulse oximeters have become a critical device in all hospitals, and they continuously monitor patients, especially those under anesthesia [7]. Recently, pulse oximeters were underscored during the pandemic breakout of SARS-CoV-2 (coronavirus disease 2019 (COVID-19)). Since COVID-19 affected the lungs, pulse oximeters were the ideal device for monitoring the respiratory status of patients suffering from COVID-19. Pulse oximeters are portable and noninvasive, allowing patients to be managed at home anywhere [9]. The pulse oximeter’s benefits are endless to modern medicine. However, some unforeseen pulse oximeter limitations have recently been discovered.

The pulse oximeter offers an easy and accurate way to measure oxygen saturation in the blood, however, studies have shown that the pulse oximeter is biased towards different skin pigmentations [15]. Those with higher melanin and a darker skin tone absorb more light from the pulse oximeters, which leads to the pulse oximeter overestimating one’s oxygen saturation [16]. This discrepancy would increase the chance of not detecting conditions such as hypoxia in patients with darker skin tones because the pulse oximeter does not give an accurate reading. This finding quickly gave rise to concerns in the medical field as not only are patients at risk but ethical discussions are raised due to racial disparities [17]. This inaccuracy by the pulse oximeter is primarily due to the exclusion of those with darker skin tones when calibrating the device. This flaw does not necessarily undermine the already reputable status of the pulse oximeter. However, it does raise concern about how the pulse oximeter could indirectly worsen a racial divide within the healthcare system [17].

## Conclusions

The modern-day pulse oximeter has come to fruition after centuries of development in medical technology, having recently revolutionized in the last 50 years. From the Lambert-Beer law as a basis to the integration of pulsatile rhythms by Takuo Aoyagi, the pulse oximeter has made significant progress allowing it to be a relevant device in medicine. However, the evolution and development of the pulse oximeter continue, as there are still things that could be improved in its design. If scientific community members do not address the pulse oximeter’s inaccuracy towards darker skin pigments, medical progress may be hindered. Ultimately, it is crucial to recognize the profound impact of the pulse oximeter on modern-day healthcare while recognizing what society can do to evolve the device further.
